# Binocular Combination of Second-Order Stimuli

**DOI:** 10.1371/journal.pone.0084632

**Published:** 2014-01-03

**Authors:** Jiawei Zhou, Rong Liu, Yifeng Zhou, Robert F. Hess

**Affiliations:** 1 CAS Key Laboratory of Brain Function and Disease, and School of Life Sciences, University of Science and Technology of China, Hefei, Anhui, PR China; 2 McGill Vision Research, Department of Ophthalmology, McGill University, Montreal, Quebec, Canada; University of Sussex, United Kingdom

## Abstract

Phase information is a fundamental aspect of visual stimuli. However, the nature of the binocular combination of stimuli defined by modulations in contrast, so-called second-order stimuli, is presently not clear. To address this issue, we measured binocular combination for first- (luminance modulated) and second-order (contrast modulated) stimuli using a binocular phase combination paradigm in seven normal adults. We found that the binocular perceived phase of second-order gratings depends on the interocular signal ratio as has been previously shown for their first order counterparts; the interocular signal ratios when the two eyes were balanced was close to 1 in both first- and second-order phase combinations. However, second-order combination is more linear than previously found for first-order combination. Furthermore, binocular combination of second-order stimuli was similar regardless of whether the carriers in the two eyes were correlated, anti-correlated, or uncorrelated. This suggests that, in normal adults, the binocular phase combination of second-order stimuli occurs after the monocular extracting of the second-order modulations. The sensory balance associated with this second-order combination can be obtained from binocular phase combination measurements.

## Introduction

One of the key aspects of primate vision is its binocularity. Even though the visual inputs to the two eyes may not be identical, binocular visual perception is always single. How the various features of the two eyes’ visual inputs combine to a single percept is an important issue in vision research. While there have been numerous studies investigating the nature of the binocular combination of features such as luminance [1]–[3], contrast [4]–[11], color [12]–[15] disparity [16]–[20], and binocular rivalry [21]–[25], little is known about the binocular combination of phase information between the two eyes. This is surprising because phase information is fundamental in identifying images [26].

Recently, Ding & Sperling introduced the dichoptic phase combination paradigm to study how the two eyes’ inputs combine [27]. In their paradigm, two horizontal sine-wave gratings with same spatial frequency and size, but equal and opposite phase-shifts (relative to the centre of the screen) were dichoptically presented to the two eyes; the perceived phase of the binocular percept was measured for a range of interocular contrast differences. The advantage of this paradigm is that an index of the two eyes’ binocular combination could be gauged in terms of the binocular perceived phase. If the visual input in one eye is more dominant than that of the other eye, the binocular perceived phase will be shifted in the direction of the phase of the grating in the dominant eye; if each eye contributes equally to binocular combination, the binocular perceived phase will be at 0°. They kept the contrast in one eye fixed and varied proportionally the contrast in the other eye in normal adults, and found that the perceived phase of the binocularly combined grating was related to the interocular contrast ratio in a non-linear way and that the two eyes were balanced when the contrast in two eyes were the same. They proposed a gain-control model to account for the binocular perceived phase vs. interocular contrast ratio function, in which the visual inputs in two eyes first go through an interocular gain-control stage and then linearly combine to provide a binocularly fused percept. This gain-control model has been successfully used in predicting the binocular perceived phase for people who have sensory imbalance, e.g., amblyopes [28]–[32] or normal adults under abnormal viewing conditions, e.g. when the two eyes have different luminances [32].

These studies have enriched our knowledge of the binocular combination of phase information. However, all of them have involved exclusively luminance-defined phase information (so-called first-order signal, i.e., the phase information derived from the local changes in luminance). This source of visual information should be distinguished from another important source of visual signals that are defined by non-luminance variation (envelope) e.g., local changes in contrast or texture [33]. Such non-luminance defined stimuli are referred to as second-order stimuli [33] and are common in natural images. Neurons in the early visual cortex respond to both first- and second-order stimuli (Baker, 1999). Previous studies have suggested that first- and second-order stimuli are processed differently [34]; the former being processed directly via linear spatiotemporal filters while the latter is processed via three sequential stages that involve a stage of linear filtering, a stage of non-linear rectification and a stage of linear filtering [34], [35]. Given the different nature in the underlying processes of first- and second-order stimuli, we address three key questions in the current study: first, *does binocular phase combination also occur for second-order stimuli;* if so, *what’s the difference between the binocular phase combination of first-order and second-order stimuli;* And finally, *does binocular combination for second-order stimuli occur before or after the extraction of second-order signals?*

To answer these questions, we assessed the two eyes’ ability to combine phase information for luminance-defined, first-order gratings and contrast-modulated, second-order gratings, using the Ding & Sperling’s dichoptic phase combination paradigm. In particular, we fixed the contrast of the first-order gratings or the modulation depth of the second-order gratings at 100% in the nondominant eye, and measured the binocularly perceived phase when the signal (the contrast of the first-order gratings or the modulation depth of the second-order gratings) in the dominant eye was varied. We found, the binocularly perceived phase of second-order stimuli was also dependent on the ratio of the interocular signal strengths, however this dependence was much more linear compared with that of first-order gratings; the interocular contrast ratio in the binocular combination of first-order stimuli and the interocular modulation ratio in the binocular combination of second-order stimuli were close to unity when the two eyes were balanced.

**Figure 1:**
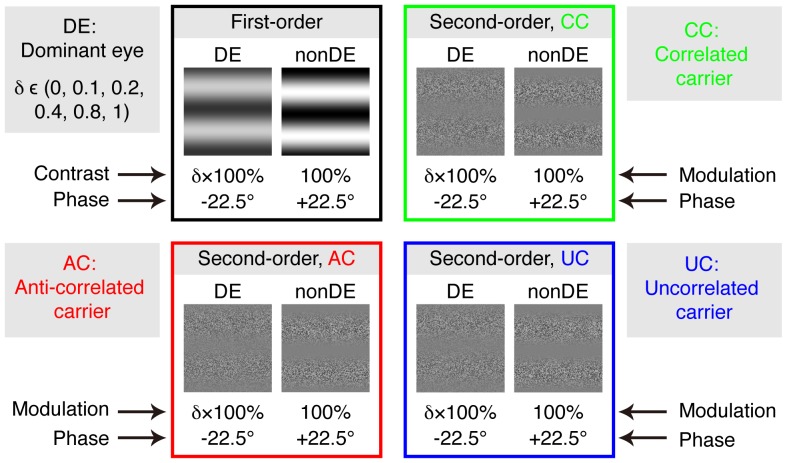
Dichoptic stimuli in the binocular phase combination test. Four dichoptic pairs were used in the test: First-order gratings, second-order gratings with correlated carriers (noise) in the two eyes, second-order gratings with anti-correlated carriers in the two eyes and second-order gratings with uncorrelated carriers in the two eyes. For each dichoptic pair, grating with fixed contrast (modulation) of 100% and phase-shift of 22.5° to one direction was inputted to the nondominant eye; grating with a proportional contrast with interocular contrast ratio of δ (δ = (0, 0.1, 0.2, 0.4, 0.8, 1)) and a reverse phase-shift by the same magnitude was inputted to the dominant eye. The phase of the binocularly combined cyclopean grating was then measured to access the two eyes contribution in binocular viewing. The perceived phase of the cyclopean grating was quantified by the half of the difference between the measured phase when the phase-shift was −22.5° in the dominant eye and +22.5° in the nondominant eye and the measured phase at the reversal configuration.

To determine whether the binocular combination occurs before or after the extraction of second-order signals, we measured the binocular phase combination using three types of second-order dichoptic pairs (**Figure 1**): the carriers in the two eyes could be correlated, anti-correlated or uncorrelated. If the extraction of second-order signals occurs after the binocular combination, we would expect quite different performance for these three second-order dichoptic pairs. Specifically, if the binocular combination involves combination of first-order signals before the extraction of the second-order envelope, then phase judgments with anti-correlated carriers should be impossible. A similar argument applies to the case of uncorrelated carriers if there is an initial stage of cross-correlation of first-order signals prior to extraction of the second-order envelope [16]. We found, however, quite similar tuning functions existed for all three second-order dichoptic pairs, which suggests that the binocular combination occurs after the monocular extraction of second-order signals.

## Materials and Methods

### Observers

Seven adults (Age: 27.3±1.4 yrs; four females) with normal or corrected to normal vision participated in our experiment. Except for the first author, all subjects were naive as to the purpose of the experiment. A written informed consent was obtained from each of them before the start of the test. The study was approved by the Institutional Review Boards of University of Science and Technology of China and McGill University.

### Apparatus

All stimuli were generated and controlled by a Mac computer running Matlab (MathWorks, Natick, MA) with PsychTool Box 3.0.9 extension [36], [37]. The stimuli were dichoptically presented with Z800 pro goggles (eMagin Corp., Washington, DC), which had a simulated viewing distance of 3.6 m, a spatial resolution of 800×600, a refresh rate of 60 HZ and a mean luminance of 160 cd/m^2^ in each eye. The advantages for using OLED micro-displays are: 1. They are linear in luminance response [38]; and 2. They exhibit pixel independence in image presentation [39]. Thus we would not expect any non-linear effects due to the display equipment [40] in our study.

### Design

A binocular phase combination paradigm [27] was used to quantitatively determine the two eye’s functional index in binocular combination with luminance-defined first-order or contrast-modulated second-order stimuli. In the test, two horizontal sine-wave gratings with equal and opposite phase-shifts of 22.5° (relative to the centre of the screen) were dichoptically presented to the two eyes. The signal strength (i.e., the contrast of first-order gratings or the modulation depth of the second-order gratings) was fixed as 100% in the nondominant eye and varied with a ratio *δ* (*δ* = [0, 0.1, 0.2, 0.4, 0.8, 1]) in the dominant eye. The phase of the binocularly combined grating was measured with an adjustment method for different interocular signal ratios (*δ*). To cancel any potential positional bias, two configurations were used in the measure: (1) the phase-shift was +22.5° in the nondominant eye and −22.5° in the dominant eye; (2) the phase-shift was −22.5° in the nondominant eye and +22.5° in the dominant eye. The perceived phase at each interocular signal ratio (*δ*) was quantified by half of the difference between the measured perceived phases in these two configurations. The two configurations at the six interocular signal strength ratios were measured eight times using constant stimuli. The perceived phase and its standard error were calculated based on these eight repetitions. The function of perceived phase versus interocular signal ratios (PvR function) was then derived for each stimuli type.

### Stimuli

The stimulus configurations were identical to that previously described [31]: two monocular horizontal sine-wave gratings with different signal strengths (i.e., luminance contrast or modulation depth) but having equal and opposite phase-shifts (relative to the centre of the screen) were dichoptically presented in the middle of the two OLED mini-displays. The sine-wave gratings had a period of 2 cycles (**Figure 1**), which subtended 6.8° of visual angle (i.e., 0.29 cycle/°). A high-contrast frame (width, 0.378°; length, 20.43°) with four white diagonal lines (width, 0.378°; length, 9.63°) was presented surrounding the grating in each eye to help observers maintain fusion. A 1-pixel black reference line was presented horizontally at the two sides of the gratings and observers were asked to move it to indicate the perceived phase after combination.

For the first-order tests, the gratings in the two eyes were defined as:
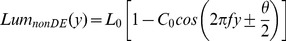
(1)

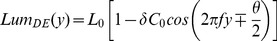
(2)


Where *L_0_* is the background luminance; *C_0_* = 100% is the base contrast in the nondominant eye; *f = *0.29 cycle/° is the spatial frequency of the gratings and *δ* is the interocular contrast ratio, *δ* = [0, 0.1, 0.2, 0.4, 0.8, 1.0]. The two dichoptic gratings in the test had an equal and opposite phase-shift of *θ/2* (relative to the centre of the screen), which was 22.5°.

For the second-order tests, the gratings in the two eyes were defined as:
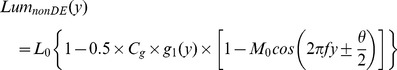
(3)

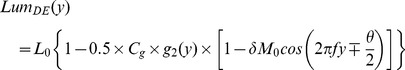
(4)


Where *L_0_* is the background luminance; *g_1_(y)* and *g_2_(y)* are the carriers in the two eyes and consisted of white noise (see **Figure 1**). For the dichoptic pair with correlated carriers, *g_1_(y)* = *g_2_(y)*; for the dichoptic pair with anti-correlated carriers, *g_1_(y)* = −*g_2_(y)*; for the dichoptic pair with uncorrelated carriers, *g_1_(y)* ≠ *g_2_(y)*. *C_g_ = *40% is the contrast of carrier; *f = *0.29 cycle/° is the spatial frequency of the envelope sine-wave gratings; *M_0_* = 100% is the base modulation depth in the nondominant eye and *δ* is the interocular modulation depth ratio, *δ* = [0, 0.1, 0.2, 0.4, 0.8, 1.0]. The two dichoptic gratings in the test had an equal and opposite phase-shift of *θ/2* (relative to the centre of the screen), which was 22.5°.

### Procedure

An alignment task was provided at the beginning of each trial to make sure the two eyes’ images were correctly fused. In the alignment task, a fixation display was presented in the centre of the larger high-contrast frame together with four white diagonal lines. This display consisted of binocular fixation crosses (3.78×3.78 degree^2^) and four monocular dots (0.378° diameter), two of which were in the 1st and 3rd quadrants in the left eye and two of which were in the 2nd and 4th quadrants in the right eye. Observers were instructed to move the image in their nondominant eye using up, down, left and right arrow keys to align the images from two eyes. After achieving stable fusion, observers were asked to press the ‘space’ key. The corresponding coordinate between two eyes was then used in the following measurement. After that, a phase adjustment procedure [30] was used to measure the perceived phase of the binocularly combined gratings. Observers were asked to adjust the position of the sided reference line to indicate the perceived phase of the cyclopean sine-wave grating, defined as the location of the centre of the dark stripe of the first-order grating or the lower contrast stripe of the second-order grating. The reference line was presented with an initial position randomly (−9 to 10 pixels) assigned relative to the centre of the frame in each trial. It was moved with a fixed step size of 1 pixel, corresponding to 4-degree phase angle of the sinewave grating. During one trial, the gratings, frames and reference lines were presented continually in the two eyes until subjects finished the phase adjustment. A typical trial lasted for about 10 seconds.

### Curve Fits

The PvR functions for different dichoptic pairs were fitted with a modified gain control model from Huang et al [30]:
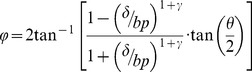
(5)


In which, *φ* is the measured perceived phase when the interocular signal strength ratio is *δ* (*δ* = [0, 0.1, 0.2, 0.4, 0.8, 1.0]); *θ* is the interocular phase difference (i.e., 45**°** in our test) and the two free parameters, *bp* and *γ*, represent the effective signal ratio at balance point (i.e., *φ* = 0°) and the non-linear factor in the binocular combination, respectively. These two free parameters jointly define the shape of the PvR function. Note that here we use a factor ‘*bp*’ to replace the attenuation/suppression factor in Huang et al [30], which is convenient in showing the zero-crossing point of the PvR function. However, our model is mathematically identical with theirs.

Curve fits were conducted in Matlab (MathWorks, Natick, MA) using nonlinear least squares method to minimized ∑(*φ_theory_* – *φ_observed_*)^2^. The goodness-of-fit was statistically tested by computing the *R-square* value:
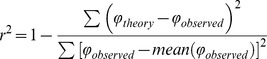
(6)


## Results

**Figure 2:**
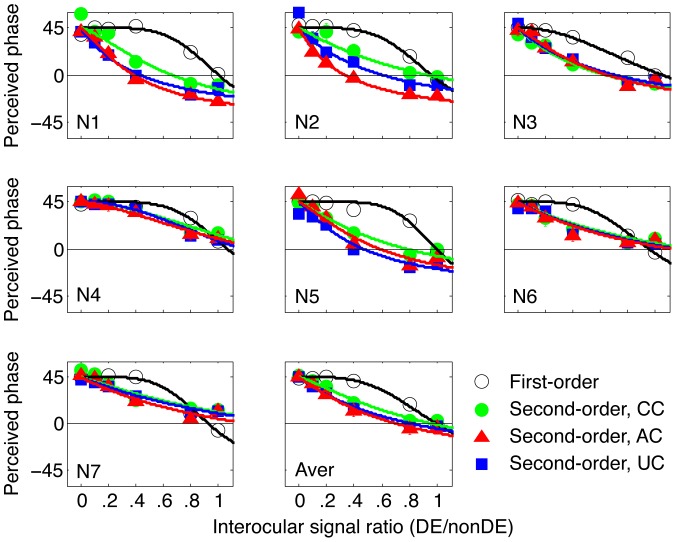
Binocularly perceived phase as a function of interocular signal ratios for the four dichoptic pairs. Results of the seven observers (N1–N7) and their averages are shown in separate panels. In each panel, the vertical axis represents perceived phase of the cyclopean grating, the horizontal axis represents interocular signal ratio (Dominant eye/nondominant eye); the four kinds of symbols represent data for four dichoptic pairs: ‘○’, first-order stimuli; ‘•’, second-order stimuli with correlated carrier in two eyes; ‘▴’, second-order stimuli with anti-correlated carries in two eyes; ‘▪’, second-order stimuli with uncorrelated stimuli. The colored solid lines represent prediction of a modified gain control model (see data fitting in the Methods part). The horizontal line in the middle of each panel indicates expected output when the perceived phase is zero. Error bars represent standard errors.

The function relating the binocularly perceived phase to the interocular signal strength ratio (PvR) for the four kinds of dichoptic pairs for each observer and their average are shown in different panels in **Figure 2**. The pattern of the PvR function that was measured with first-order gratings is quite similar to that observed previously [27]–[32], in which, the binocularly perceived phase decreased, in a nonlinear fashion, from +45° to near 0°, as the interocular contrast ratio increased from 0 to 1. For second-order dichoptic stimuli, the binocularly perceived phase also decreased from +45° to near 0° as the interocular contrast ratio increased from 0 to 1, however it decreased in a more linear fashion compared with that of its first-order counterpart. This pattern of PvR function was similar for all three second-order dichoptic pairs and was not significantly dependent on the carrier type (F(2,18) = 1.036, *p* = 0.375).

Since the balance point (*bp*) and the linearity (***γ***) jointly define the shape of the PvR, we quantitatively assessed any difference in the PvR for different stimuli by calculating these two critical parameters based on the gain-control model (see Curve fits in Method). The predictions of the model are drawn with solid lines in Figure 2. In summary, the model successfully accounted for 97.9%, 95.1%, 96.1% and 95.8% of the variance in phase combination of first-order gratings and second-order grating with correlated carriers, anti-correlated carriers and uncorrelated carriers, respectively. For the average observer (i.e., the last panel in **Figure 2**), the model also successfully accounted for 99.4%, 98.5%, 99.1% and 99.1% of the variance respectively for these four dichoptic pairs, respectively.

**Figure 3:**
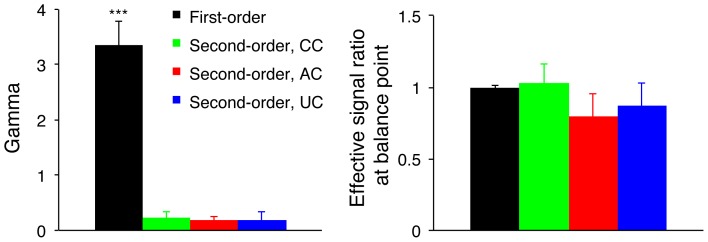
The averaged fitted parameters of the four dichoptic pairs. Left panel shows the averaged ‘Gamma’ of the four dichoptic pairs. ‘Gamma’ denotes the non-linearity in the binocular combination, the less the gamma, the less the non-linearity; Right panel shows the averaged effective signal ratio at balance point of the four dichoptic pairs. Effective signal ratio at balance point indicates the signal ratio that is needed to balance the dominant eye with the nondominant eye in binocular combination. Error bars represent standard errors. Significance of the compartment between the second-order dichoptic pairs and first-order dichoptic pair is marked in the figure: ***, *p*<0.001, 2-tailed T test.

In **Figure 3**, the averaged non-linear factor ‘Gamma’ (**Figure 3**, left panel) and the effective signal ratio at balance point ‘*bp*’ (**Figure 3**, right panel) that were fitted by the gain-control model are plotted for the four dichoptic pairs, respectively. ‘Gamma’ denotes the degree of non-linearity in the binocular combination, the less the ‘Gamma’, the less the non-linearity [27]. Averaged across the three second-order dichoptic pairs, the non-linear factor ‘Gamma’ was 0.19±0.065 (Mean ± S.E.M), much less than that of the first-order dichoptic pair (all t(6)>7.7, *p*<0.001, Paired sample T-test, 2-tailed), which was 3.34±0.43. Such linearity in binocular phase combination occurred in all the three carrier types and was not significantly different between each type (all t(6)<0.74, *p*>0.48, Paired samples T-test, 2-tailed), ruling out any possible influences of the carriers themselves.

The effective signal ratio at balance point ‘*bp*’ indicates the interocular signal ratio that is needed for the nondominant eye to produce a balanced combination with that of the dominant eye, i.e., the zero-crossing point of the PvR function. The magnitude of the effective signal ratio at balance point reflects the sensory imbalance between eyes, the closer to 1, the less the imbalance [30]. We found that the effective signal ratios at balance point at all the first-order and second-order dichoptic pairs were not significantly different from 1 (all t(6)<1.32, *p*>0.23, 2-tailed), and the effective signal ratios at balance point for all the three second-order dichoptic pairs were not significantly different from that of the first-order dichoptic pair (all t(6)<1.28, *p*>0.24, 2-tailed). These results indicate that the two eyes of normal adults are balanced at similar ranges of interocular signal ratios for both first-order and second-order signals. However, one should note that the effective signal ratios at balance point were not identical for the three second-order dichoptic pairs; the correlated carrier condition had a significantly larger balance point than that of the other two carrier conditions (correlated carrier condition vs. anti-correlated carrier condition: t(6) = 2.90, *p* = 0.027, 2-tailed; correlated carrier condition vs. uncorrelated carrier condition: t(6) = 2.81, *p* = 0.031, 2-tailed). This is not surprising since different carriers in the two eyes could induce additional interocular imbalance resulting in higher sensory imbalance in binocular combination.

**Figure 4:**
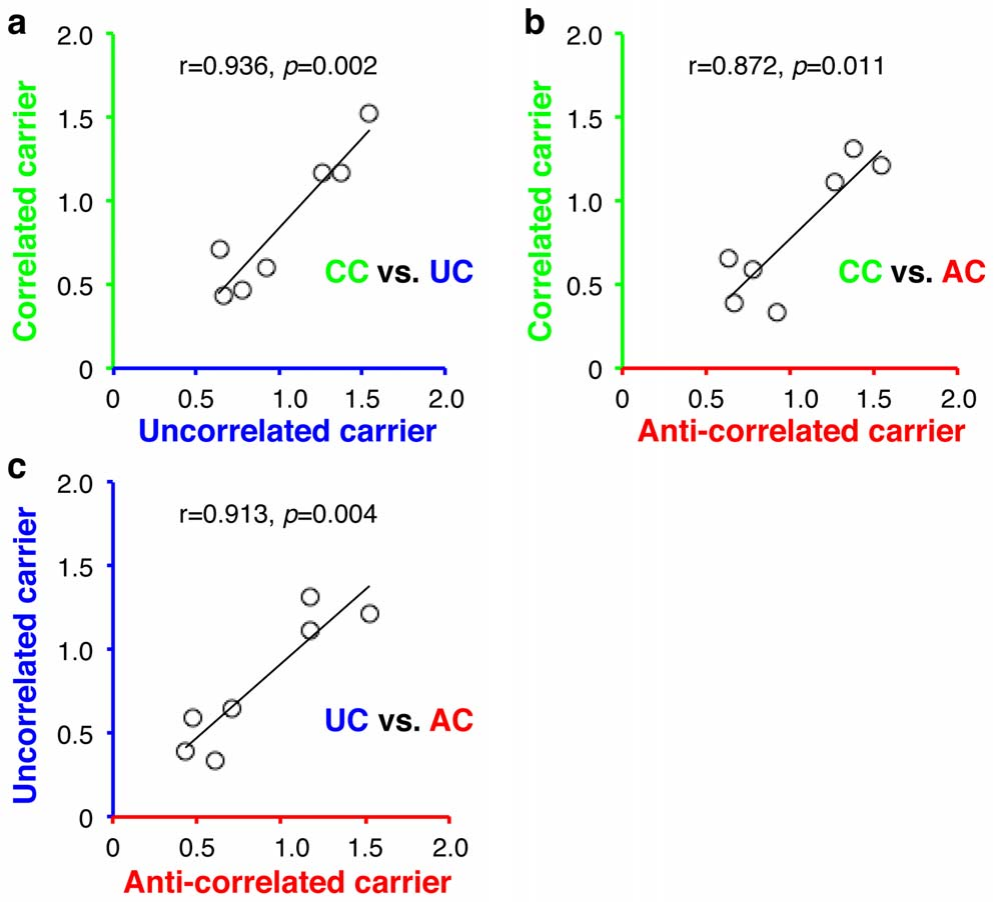
Correlations of the effective signal ratios at the balance point between the three second-order dichoptic pairs. a) Correlated carrier vs. uncorrelated carrier; b) Correlated carrier vs. anti-correlated carrier; c) Uncorrelated carrier vs. anti-correlated carrier. In each panel, different dots represent different subjects; the solid line represents a linear fitting.

Even though the two eyes of normal adults were balanced at a similar range of interocular signal ratios with both the first- and second-order dichoptic pairs, the effective signal ratios at the balance point were not significantly correlated between them (all *p*>0.45, Paired samples correlation, 2-tailed). However, significant correlations were found among the three second-order dichoptic pairs (Figure 4, all *p<*0.012, 2-tailed), further confirming that the binocular combination of second-order stimuli was not governed by the carrier-correlations in the two eyes.

## Discussion

We have shown that the binocularly perceived phase of second-order signals is also dependent on the interocular contrast ratio; in a normal binocular individual, the two eyes are balanced when the signal strengths are equal in two eyes, this is true for both first-order and second-order signals. Furthermore, the phase vs. interocular signal ratios curves for the three second-order dichoptic pairs are quite similar with each other, no matter whether the carriers in the two eyes are correlated, anti-correlated or uncorrelated. The PvR functions for second-order stimuli could be predicted by the gain-control model [27] assuming that at a preceding monocular stage the second-order modulations are extracted. However, in the case of second-order signals, the gain-control is much more linear compared with that of first-order signals.

One technical concern is the possible influence of luminance artifacts in our second-order stimuli. The first possible source of first-order artifacts comes from the non-linearities in a CRT display. Previous reports have shown that even for the gamma-corrected CRT monitor, there could still be some adjacent pixel non-linear effects [40]. In our study, however, we used an OLED display instead of the CRT monitor, which is linear in its luminance response [38] and exhibits pixel-independence [39]. Thus we would not expect any luminance non-linearity due to the display equipment we used in our study. The second possible source of first-order artifacts results from local DC noise bias in noise carriers [41], or the side-band spectral components of some second-order stimuli that may be detected by first-order channels, the so-called side-band effect [42]. These kinds of first-order artifacts have been demonstrated to exist in second-order stimuli with low pass (e.g., 1/f) noise carriers and high spatial frequency modulations, but not in second-order stimuli with broadband and high pass noise carriers [43]. Since a broadband noise carrier and a quite low frequency modulation (i.e., 0.29 cycle/°) were used in our study, our stimulus was exempt from this possible artifact. The third possible source of first-order artifacts is a nonlinearity in the receptors [44]. We argue that, this artifact, even if present for our task, must be too weak to produce any significant effect on the binocular phase measurement with second-order stimuli. Previous studies of binocular phase combination [27], [30], [45] have shown that the binocular perceived phase has a non-linear relationship with the interocular contrast ratio of first-order stimuli, even when the contrast is quite low (i.e., around 5%). In other words, even if there was a low contrast first-order artifact generated within the visual system (or indeed in our display) the influence it would have on the phase measurement is not able to explain our main result, that of a linear relationship. Therefore we are confident that our main conclusion is not the result of first-order luminance artifacts but of second-order processing per se.

To our knowledge, this is the first study that has investigated binocular combination of suprathreshold contrast-modulated stimuli. There are several previous reports in the literature on the binocular summation of the second-order signals for contrast detection [46] and the second-order stereopsis [16]. For contrast detection, Georgeson and Schofield [46] found that the binocular summation produced a facilitation of about 5–6 dB for contrast-modulated second-order signals, which was quite close to the prediction of linear summation. The idea of linear summation for contrast detection is similar to what we obtained here in our study for the combination of suprathreshold stimuli using the phase combination paradigm. The current theories governing the binocular summation of first-order stimuli are in agreement with the existing of non-linear gain-control in interocular interaction [27], [45], [47], [48]. The linearity in binocular phase combination shown by suprathreshold second-order signals and non-linearity in binocular phase combination shown by first-order signals suggests that the interocular gain-control of first- and second-order stimuli might not be the result of the same mechanism. This speculation gains some support from our findings in comparing the relationship of the effective contrast ratios at the balance point between the first- and second-order stimuli, in which, they were similar but not significantly correlated.

A key finding in Georgeson and Schofield’s study is that the facilitation in detecting second-order signals occurs no matter whether the carriers in the two eyes were correlated, un-correlated, or anti-correlated. They concluded that the binocular summation arose from the summation of envelope responses. Their findings are consistent with our results, since both studies show that the binocular combination of second-order signals is similar no matter whether the two eyes have the same or different noise. Similar observations were also made by Wilcox and Hess [16]. In measuring second-order stereopsis, they found that stereo acuity was quite similar no matter whether the carriers in the two eyes were correlated or uncorrelated. Our results, together with these previous reports, suggest that the binocular combination for contrast-modulated second-order signals occurs after the monocular extraction of second-order envelopes. This general conclusion agrees with the second-stage convergence model that was proposed for cat area 18 in processing stereopsis from second-order contrast cues (see Figure 5B in [49] in which, it was proposed that second-order signals were monocularly processed and then combined binocularly at a second-stage neurons).
